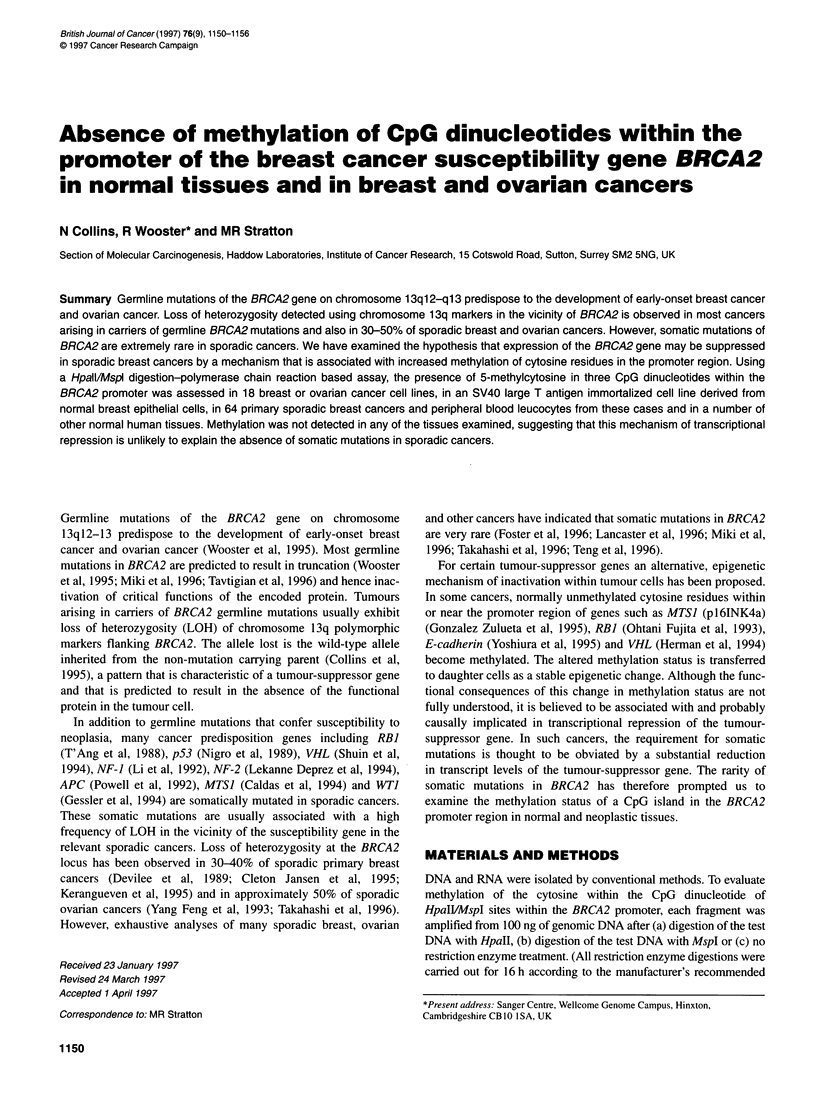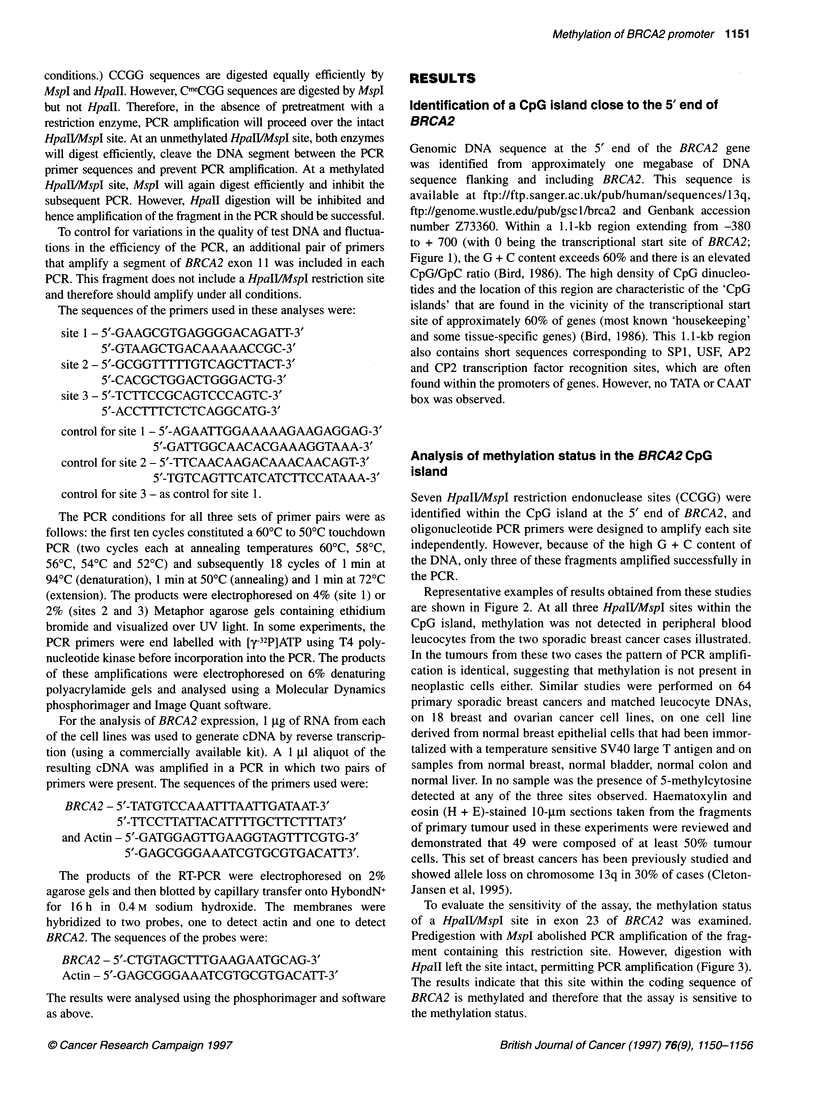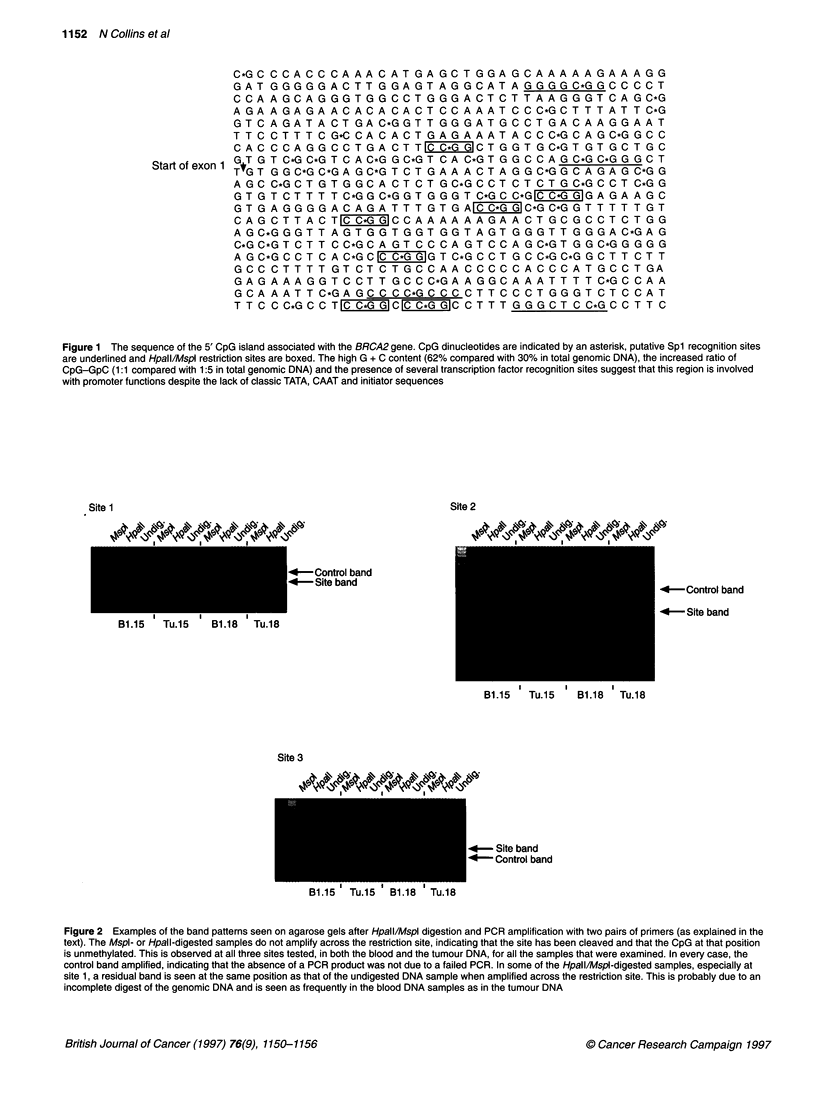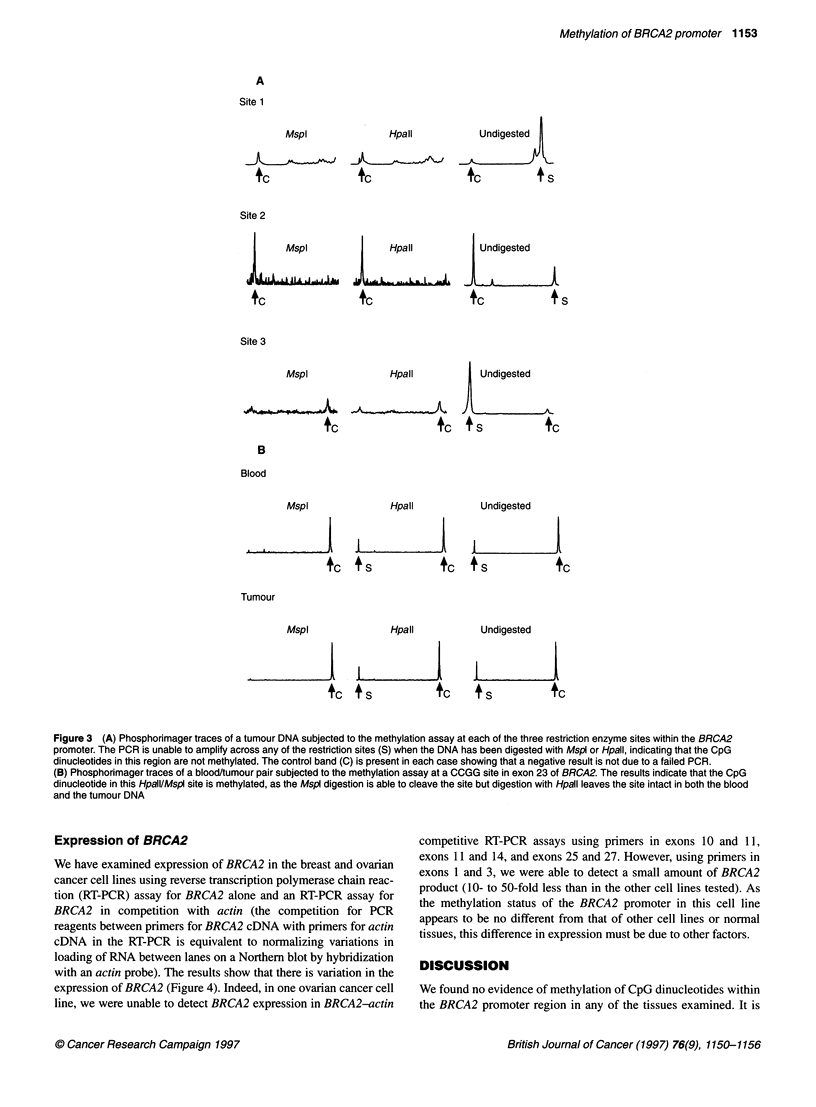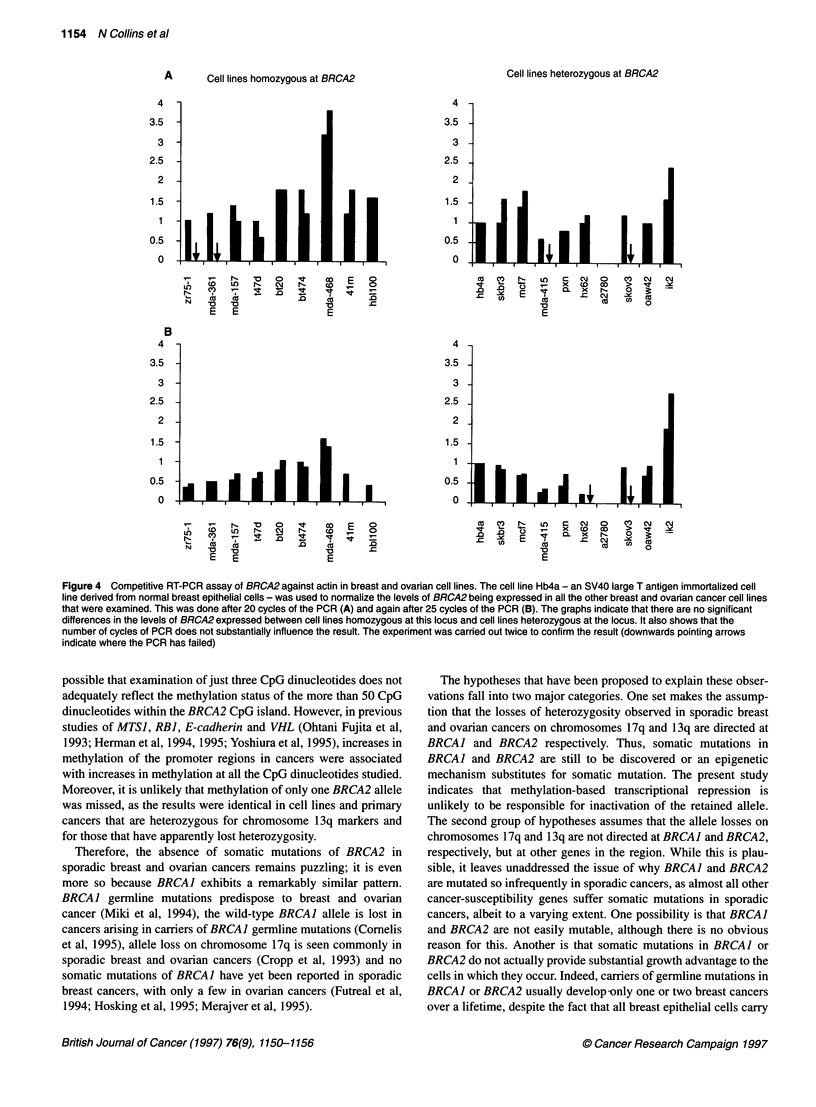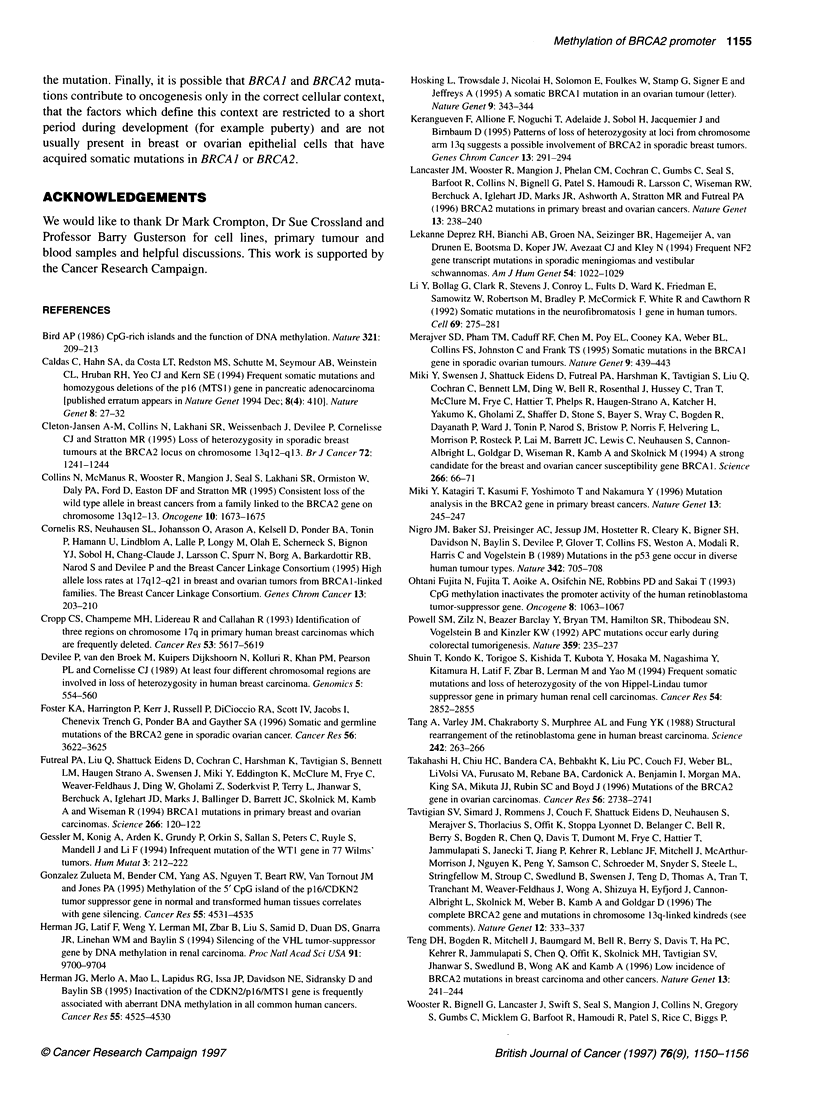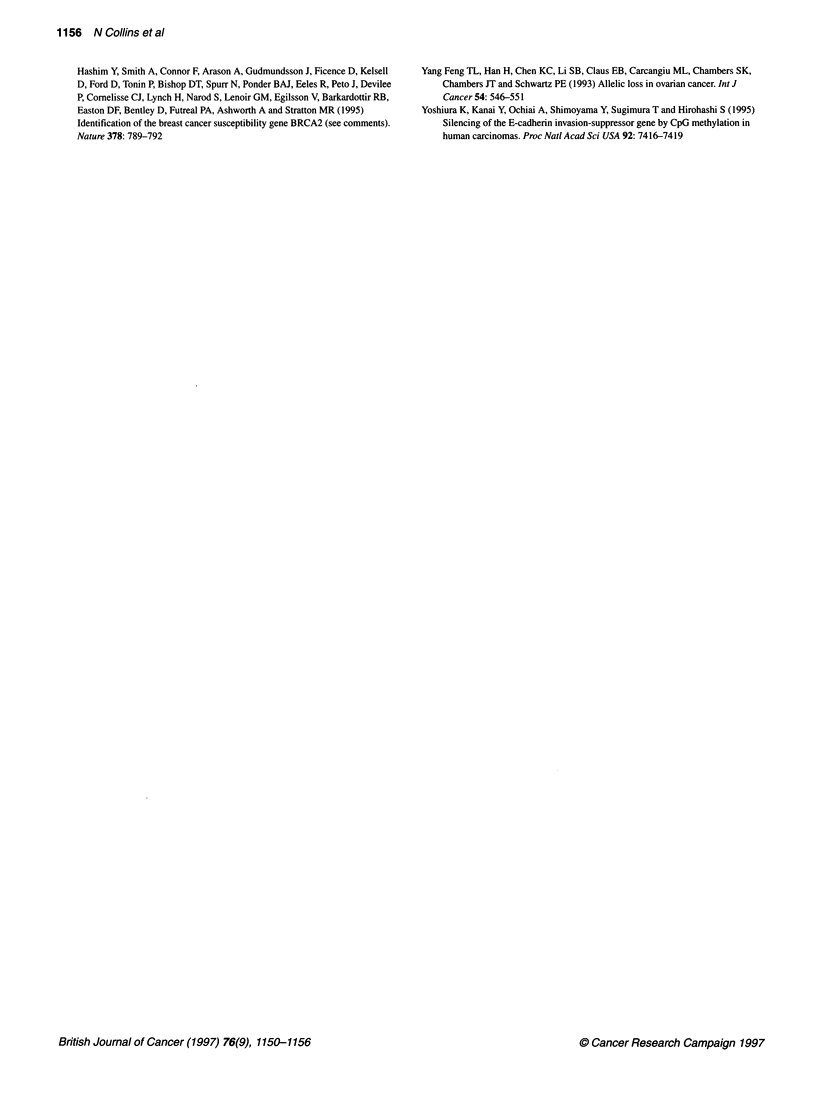# Absence of methylation of CpG dinucleotides within the promoter of the breast cancer susceptibility gene BRCA2 in normal tissues and in breast and ovarian cancers.

**DOI:** 10.1038/bjc.1997.526

**Published:** 1997

**Authors:** N. Collins, R. Wooster, M. R. Stratton

**Affiliations:** Section of Molecular Carcinogenesis, Haddow Laboratories, Institute of Cancer Research, Sutton, Surrey, UK.

## Abstract

**Images:**


British Journal of Cancer (1997) 76(9), 1150–1156
© 1997 Cancer Research Campaign

### Absence of methylation of CpG dinucleotides within the promoter of the breast cancer susceptibility gene *BRCA2* in normal tissues and in breast and ovarian cancers

**N Collins, R Wooster* and MR Stratton**

Section of Molecular Carcinogenesis, Haddow Laboratories, Institute of Cancer Research, 15 Cotswold Road, Sutton, Surrey SM2 5NG, UK

**Summary** Germline mutations of the *BRCA2* gene on chromosome 13q12–q13 predispose to the development of early-onset breast cancer and ovarian cancer. Loss of heterozygosity detected using chromosome 13q markers in the vicinity of *BRCA2* is observed in most cancers arising in carriers of germline *BRCA2* mutations and also in 30–50% of sporadic breast and ovarian cancers. However, somatic mutations of *BRCA2* are extremely rare in sporadic cancers. We have examined the hypothesis that expression of the *BRCA2* gene may be suppressed in sporadic breast cancers by a mechanism that is associated with increased methylation of cytosine residues in the promoter region. Using a *HpaII/MspI* digestion–polymerase chain reaction based assay, the presence of 5-methylcytosine in three CpG dinucleotides within the *BRCA2* promoter was assessed in 18 breast or ovarian cancer cell lines, in an SV40 large T antigen immortalized cell line derived from normal breast epithelial cells, in 64 primary sporadic breast cancers and peripheral blood leucocytes from these cases and in a number of other normal human tissues. Methylation was not detected in any of the tissues examined, suggesting that this mechanism of transcriptional repression is unlikely to explain the absence of somatic mutations in sporadic cancers.

Germline mutations of the *BRCA2* gene on chromosome 13q12–13 predispose to the development of early-onset breast cancer and ovarian cancer (Wooster et al, 1995). Most germline mutations in *BRCA2* are predicted to result in truncation (Wooster et al, 1995; Miki et al, 1996; Tavtigian et al, 1996) and hence inactivation of critical functions of the encoded protein. Tumours arising in carriers of *BRCA2* germline mutations usually exhibit loss of heterozygosity (LOH) of chromosome 13q polymorphic markers flanking *BRCA2*. The allele lost is the wild-type allele inherited from the non-mutation carrying parent (Collins et al, 1995), a pattern that is characteristic of a tumour-suppressor gene and that is predicted to result in the absence of the functional protein in the tumour cell.

In addition to germline mutations that confer susceptibility to neoplasia, many cancer predisposition genes including *RB1* (T'Ang et al, 1988), *p53* (Nigro et al, 1989), *VHL* (Shuin et al, 1994), *NF-1* (Li et al, 1992), *NF-2* (Lekanne Deprez et al, 1994), *APC* (Powell et al, 1992), *MTS1* (Caldas et al, 1994) and *WT1* (Gessler et al, 1994) are somatically mutated in sporadic cancers. These somatic mutations are usually associated with a high frequency of LOH in the vicinity of the susceptibility gene in the relevant sporadic cancers. Loss of heterozygosity at the *BRCA2* locus has been observed in 30–40% of sporadic primary breast cancers (Devilee et al, 1989; Cleton Jansen et al, 1995; Kerangueven et al, 1995) and in approximately 50% of sporadic ovarian cancers (Yang Feng et al, 1993; Takahashi et al, 1996). However, exhaustive analyses of many sporadic breast, ovarian and other cancers have indicated that somatic mutations in *BRCA2* are very rare (Foster et al, 1996; Lancaster et al, 1996; Miki et al, 1996; Takahashi et al, 1996; Teng et al, 1996).

For certain tumour-suppressor genes an alternative, epigenetic mechanism of inactivation within tumour cells has been proposed. In some cancers, normally unmethylated cytosine residues within or near the promoter region of genes such as *MTS1* (p16INK4a) (Gonzalez Zulueta et al, 1995), *RB1* (Ohtani Fujita et al, 1993), *E-cadherin* (Yoshiura et al, 1995) and *VHL* (Herman et al, 1994) become methylated. The altered methylation status is transferred to daughter cells as a stable epigenetic change. Although the functional consequences of this change in methylation status are not fully understood, it is believed to be associated with and probably causally implicated in transcriptional repression of the tumour-suppressor gene. In such cancers, the requirement for somatic mutations is thought to be obviated by a substantial reduction in transcript levels of the tumour-suppressor gene. The rarity of somatic mutations in *BRCA2* has therefore prompted us to examine the methylation status of a CpG island in the *BRCA2* promoter region in normal and neoplastic tissues.

### MATERIALS AND METHODS

DNA and RNA were isolated by conventional methods. To evaluate methylation of the cytosine within the CpG dinucleotide of *HpaII/MspI* sites within the *BRCA2* promoter, each fragment was amplified from 100 ng of genomic DNA after (a) digestion of the test DNA with *HpaII*, (b) digestion of the test DNA with *MspI* or (c) no restriction enzyme treatment. (All restriction enzyme digestions were carried out for 16 h according to the manufacturer's recommended

*Received 23 January 1997*
*Revised 24 March 1997*
*Accepted 1 April 1997*

*Correspondence to:* MR Stratton

**Present address:* Sanger Centre, Wellcome Genome Campus, Hinxton, Cambridgeshire CB10 1SA, UK

conditions.) CCGG sequences are digested equally efficiently by *Msp*I and *Hpa*II. However, C$^{me}$CGG sequences are digested by *Msp*I but not *Hpa*II. Therefore, in the absence of pretreatment with a restriction enzyme, PCR amplification will proceed over the intact *Hpa*II/*Msp*I site. At an unmethylated *Hpa*II/*Msp*I site, both enzymes will digest efficiently, cleave the DNA segment between the PCR primer sequences and prevent PCR amplification. At a methylated *Hpa*II/*Msp*I site, *Msp*I will again digest efficiently and inhibit the subsequent PCR. However, *Hpa*II digestion will be inhibited and hence amplification of the fragment in the PCR should be successful.

To control for variations in the quality of test DNA and fluctuations in the efficiency of the PCR, an additional pair of primers that amplify a segment of *BRCA2* exon 11 was included in each PCR. This fragment does not include a *Hpa*II/*Msp*I restriction site and therefore should amplify under all conditions.

The sequences of the primers used in these analyses were:

site 1 – 5′-GAAGCGTGAGGGGACAGATT-3′
5′-GTAAGCTGACAAAAACCGC-3′
site 2 – 5′-GCGGTTTTTGTCAGCTTACT-3′
5′-CACGCTGGACTGGGACTG-3′
site 3 – 5′-TCTTCCGCAGTCCCAGTC-3′
5′-ACCTTTCTCTCAGGCATG-3′

control for site 1 – 5′-AGAATTGGAAAAAGAAGAGGAG-3′
5′-GATTGGCAACACGAAAGGTAAA-3′
control for site 2 – 5′-TTCAACAAGACAAACAACAGT-3′
5′-TGTCAGTTCATCATCTTCCATAAA-3′
control for site 3 – as control for site 1.

The PCR conditions for all three sets of primer pairs were as follows: the first ten cycles constituted a 60°C to 50°C touchdown PCR (two cycles each at annealing temperatures 60°C, 58°C, 56°C, 54°C and 52°C) and subsequently 18 cycles of 1 min at 94°C (denaturation), 1 min at 50°C (annealing) and 1 min at 72°C (extension). The products were electrophoresed on 4% (site 1) or 2% (sites 2 and 3) Metaphor agarose gels containing ethidium bromide and visualized over UV light. In some experiments, the PCR primers were end labelled with [$\gamma$-$^{32}$P]ATP using T4 polynucleotide kinase before incorporation into the PCR. The products of these amplifications were electrophoresed on 6% denaturing polyacrylamide gels and analysed using a Molecular Dynamics phosphorimager and Image Quant software.

For the analysis of *BRCA2* expression, 1 µg of RNA from each of the cell lines was used to generate cDNA by reverse transcription (using a commercially available kit). A 1 µl aliquot of the resulting cDNA was amplified in a PCR in which two pairs of primers were present. The sequences of the primers used were:

*BRCA2* – 5′-TATGTCCAAATTTAATTGATAAT-3′
5′-TTCCTTATTACATTTTGCTTCTTTAT3′
and Actin – 5′-GATGGAGTTGAAGGTAGTTTCGTG-3′
5′-GAGCGGGAAATCGTGCGTGACATT3′.

The products of the RT-PCR were electrophoresed on 2% agarose gels and then blotted by capillary transfer onto HybondN+ for 16 h in 0.4 M sodium hydroxide. The membranes were hybridized to two probes, one to detect actin and one to detect *BRCA2*. The sequences of the probes were:

*BRCA2* – 5′-CTGTAGCTTTGAAGAATGCAG-3′
Actin – 5′-GAGCGGGAAATCGTGCGTGACATT-3′

The results were analysed using the phosphorimager and software as above.

### RESULTS

### Identification of a CpG island close to the 5′ end of *BRCA2*

Genomic DNA sequence at the 5′ end of the *BRCA2* gene was identified from approximately one megabase of DNA sequence flanking and including *BRCA2*. This sequence is available at ftp://ftp.sanger.ac.uk/pub/human/sequences/13q, ftp://genome.wustle.edu/pub/gsc1/brca2 and Genbank accession number Z73360. Within a 1.1-kb region extending from –380 to + 700 (with 0 being the transcriptional start site of *BRCA2*; Figure 1), the G + C content exceeds 60% and there is an elevated CpG/GpC ratio (Bird, 1986). The high density of CpG dinucleotides and the location of this region are characteristic of the 'CpG islands' that are found in the vicinity of the transcriptional start site of approximately 60% of genes (most known 'housekeeping' and some tissue-specific genes) (Bird, 1986). This 1.1-kb region also contains short sequences corresponding to SP1, USF, AP2 and CP2 transcription factor recognition sites, which are often found within the promoters of genes. However, no TATA or CAAT box was observed.

### Analysis of methylation status in the *BRCA2* CpG island

Seven *Hpa*II/*Msp*I restriction endonuclease sites (CCGG) were identified within the CpG island at the 5′ end of *BRCA2*, and oligonucleotide PCR primers were designed to amplify each site independently. However, because of the high G + C content of the DNA, only three of these fragments amplified successfully in the PCR.

Representative examples of results obtained from these studies are shown in Figure 2. At all three *Hpa*II/*Msp*I sites within the CpG island, methylation was not detected in peripheral blood leucocytes from the two sporadic breast cancer cases illustrated. In the tumours from these two cases the pattern of PCR amplification is identical, suggesting that methylation is not present in neoplastic cells either. Similar studies were performed on 64 primary sporadic breast cancers and matched leucocyte DNAs, on 18 breast and ovarian cancer cell lines, on one cell line derived from normal breast epithelial cells that had been immortalized with a temperature sensitive SV40 large T antigen and on samples from normal breast, normal bladder, normal colon and normal liver. In no sample was the presence of 5-methylcytosine detected at any of the three sites observed. Haematoxylin and eosin (H + E)-stained 10-µm sections taken from the fragments of primary tumour used in these experiments were reviewed and demonstrated that 49 were composed of at least 50% tumour cells. This set of breast cancers has been previously studied and showed allele loss on chromosome 13q in 30% of cases (Cleton-Jansen et al, 1995).

To evaluate the sensitivity of the assay, the methylation status of a *Hpa*II/*Msp*I site in exon 23 of *BRCA2* was examined. Predigestion with *Msp*I abolished PCR amplification of the fragment containing this restriction site. However, digestion with *Hpa*II left the site intact, permitting PCR amplification (Figure 3). The results indicate that this site within the coding sequence of *BRCA2* is methylated and therefore that the assay is sensitive to the methylation status.

© Cancer Research Campaign 1997

```
                 C*GCCCACCCAAACATGAGCTGGAGCAAAAAGAAAGG
                 GATGGGGGACTTGGAGTAGGCATAGGGGC*GGCCCCT
                 CCAAGCAGGGTGGCCTGGGACTCTTAAGGGTCAGC*G
                 AGAAGAGAACACACACTCCAAATCCC*GCTTTATTC*G
                 GTCAGATACTGAC*GGTTGGGATGCCTGACAAGGAAT
                 TTCCTTTCG*CCACACTGAGAAATACCC*GCAGC*GGCC
                 CACCCAGGCCTGACTTCC*GGCTGGTGC*GTGTGCTGC
                 GTGTC*GC*GTCAC*GGC*GTCAC*GTGGCCAGC*GC*GGGCT
Start of exon 1  T▼GTGGC*GC*GAGC*GTCTGAAACTAGGC*GGCAGAGC*GG
                 AGCC*GCTGTGGCACTCTGC*GCCTCTCTGC*GCCTC*GG
                 GTGTCTTTTC*GGC*GGTGGGTC*GCC*GCC*GGGAGAAGC
                 GTGAGGGGACAGATTTGTGACC*GGC*GC*GGTTTTTGT
                 CAGCTTACTCC*GGCCAAAAAAAGAACTGCGCCTCTGG
                 AGC*GGGTTAGTGGTGGTGGTAGTGGGTTGGGAC*GAG
                 C*GC*GTCTTCC*GCAGTCCCAGTCCAGC*GTGGC*GGGGG
                 AGC*GCCTCAC*GCCC*GGGTC*GCCTGCC*GC*GGCTTCTT
                 GCCCTTTTGTCTCTGCCAACCCCCACCCATGCCTGA
                 GAGAAAGGTCCTTGCCC*GAAGGCAAATTTTC*GCCAA
                 GCAAATTC*GAGCCCC*GCCCCTTCCCTGGGTCTCCAT
                 TTCCC*GCCTCC*GGCCC*GGCCTTTGGGCTCC*GCCTTC
```

**Figure 1** The sequence of the 5′ CpG island associated with the *BRCA2* gene. CpG dinucleotides are indicated by an asterisk, putative Sp1 recognition sites are underlined and *Hpa*II/*Msp*I restriction sites are boxed. The high G + C content (62% compared with 30% in total genomic DNA), the increased ratio of CpG–GpC (1:1 compared with 1:5 in total genomic DNA) and the presence of several transcription factor recognition sites suggest that this region is involved with promoter functions despite the lack of classic TATA, CAAT and initiator sequences

**Figure 2** Examples of the band patterns seen on agarose gels after *Hpa*II/*Msp*I digestion and PCR amplification with two pairs of primers (as explained in the text). The *Msp*I- or *Hpa*II-digested samples do not amplify across the restriction site, indicating that the site has been cleaved and that the CpG at that position is unmethylated. This is observed at all three sites tested, in both the blood and the tumour DNA, for all the samples that were examined. In every case, the control band amplified, indicating that the absence of a PCR product was not due to a failed PCR. In some of the *Hpa*II/*Msp*I-digested samples, especially at site 1, a residual band is seen at the same position as that of the undigested DNA sample when amplified across the restriction site. This is probably due to an incomplete digest of the genomic DNA and is seen as frequently in the blood DNA samples as in the tumour DNA

*© Cancer Research Campaign 1997*

**Figure 3** (**A**) Phosphorimager traces of a tumour DNA subjected to the methylation assay at each of the three restriction enzyme sites within the *BRCA2* promoter. The PCR is unable to amplify across any of the restriction sites (S) when the DNA has been digested with *Msp*I or *Hpa*II, indicating that the CpG dinucleotides in this region are not methylated. The control band (C) is present in each case showing that a negative result is not due to a failed PCR. (**B**) Phosphorimager traces of a blood/tumour pair subjected to the methylation assay at a CCGG site in exon 23 of *BRCA2*. The results indicate that the CpG dinucleotide in this *Hpa*II/*Msp*I site is methylated, as the *Msp*I digestion is able to cleave the site but digestion with *Hpa*II leaves the site intact in both the blood and the tumour DNA

### Expression of *BRCA2*

We have examined expression of *BRCA2* in the breast and ovarian cancer cell lines using reverse transcription polymerase chain reaction (RT-PCR) assay for *BRCA2* alone and an RT-PCR assay for *BRCA2* in competition with *actin* (the competition for PCR reagents between primers for *BRCA2* cDNA with primers for *actin* cDNA in the RT-PCR is equivalent to normalizing variations in loading of RNA between lanes on a Northern blot by hybridization with an *actin* probe). The results show that there is variation in the expression of *BRCA2* (Figure 4). Indeed, in one ovarian cancer cell line, we were unable to detect *BRCA2* expression in *BRCA2–actin* competitive RT-PCR assays using primers in exons 10 and 11, exons 11 and 14, and exons 25 and 27. However, using primers in exons 1 and 3, we were able to detect a small amount of *BRCA2* product (10- to 50-fold less than in the other cell lines tested). As the methylation status of the *BRCA2* promoter in this cell line appears to be no different from that of other cell lines or normal tissues, this difference in expression must be due to other factors.

### DISCUSSION

We found no evidence of methylation of CpG dinucleotides within the *BRCA2* promoter region in any of the tissues examined. It is

© Cancer Research Campaign 1997 

**Figure 4** Competitive RT-PCR assay of *BRCA2* against actin in breast and ovarian cell lines. The cell line Hb4a – an SV40 large T antigen immortalized cell line derived from normal breast epithelial cells – was used to normalize the levels of *BRCA2* being expressed in all the other breast and ovarian cancer cell lines that were examined. This was done after 20 cycles of the PCR (**A**) and again after 25 cycles of the PCR (**B**). The graphs indicate that there are no significant differences in the levels of *BRCA2* expressed between cell lines homozygous at this locus and cell lines heterozygous at the locus. It also shows that the number of cycles of PCR does not substantially influence the result. The experiment was carried out twice to confirm the result (downwards pointing arrows indicate where the PCR has failed)

possible that examination of just three CpG dinucleotides does not adequately reflect the methylation status of the more than 50 CpG dinucleotides within the *BRCA2* CpG island. However, in previous studies of *MTS1*, *RB1*, *E-cadherin* and *VHL* (Ohtani Fujita et al, 1993; Herman et al, 1994, 1995; Yoshiura et al, 1995), increases in methylation of the promoter regions in cancers were associated with increases in methylation at all the CpG dinucleotides studied. Moreover, it is unlikely that methylation of only one *BRCA2* allele was missed, as the results were identical in cell lines and primary cancers that are heterozygous for chromosome 13q markers and for those that have apparently lost heterozygosity.

Therefore, the absence of somatic mutations of *BRCA2* in sporadic breast and ovarian cancers remains puzzling; it is even more so because *BRCA1* exhibits a remarkably similar pattern. *BRCA1* germline mutations predispose to breast and ovarian cancer (Miki et al, 1994), the wild-type *BRCA1* allele is lost in cancers arising in carriers of *BRCA1* germline mutations (Cornelis et al, 1995), allele loss on chromosome 17q is seen commonly in sporadic breast and ovarian cancers (Cropp et al, 1993) and no somatic mutations of *BRCA1* have yet been reported in sporadic breast cancers, with only a few in ovarian cancers (Futreal et al, 1994; Hosking et al, 1995; Merajver et al, 1995).

The hypotheses that have been proposed to explain these observations fall into two major categories. One set makes the assumption that the losses of heterozygosity observed in sporadic breast and ovarian cancers on chromosomes 17q and 13q are directed at *BRCA1* and *BRCA2* respectively. Thus, somatic mutations in *BRCA1* and *BRCA2* are still to be discovered or an epigenetic mechanism substitutes for somatic mutation. The present study indicates that methylation-based transcriptional repression is unlikely to be responsible for inactivation of the retained allele. The second group of hypotheses assumes that the allele losses on chromosomes 17q and 13q are not directed at *BRCA1* and *BRCA2*, respectively, but at other genes in the region. While this is plausible, it leaves unaddressed the issue of why *BRCA1* and *BRCA2* are mutated so infrequently in sporadic cancers, as almost all other cancer-susceptibility genes suffer somatic mutations in sporadic cancers, albeit to a varying extent. One possibility is that *BRCA1* and *BRCA2* are not easily mutable, although there is no obvious reason for this. Another is that somatic mutations in *BRCA1* or *BRCA2* do not actually provide substantial growth advantage to the cells in which they occur. Indeed, carriers of germline mutations in *BRCA1* or *BRCA2* usually develop only one or two breast cancers over a lifetime, despite the fact that all breast epithelial cells carry

*© Cancer Research Campaign 1997*

the mutation. Finally, it is possible that *BRCA1* and *BRCA2* mutations contribute to oncogenesis only in the correct cellular context, that the factors which define this context are restricted to a short period during development (for example puberty) and are not usually present in breast or ovarian epithelial cells that have acquired somatic mutations in *BRCA1* or *BRCA2*.

### ACKNOWLEDGEMENTS

We would like to thank Dr Mark Crompton, Dr Sue Crossland and Professor Barry Gusterson for cell lines, primary tumour and blood samples and helpful discussions. This work is supported by the Cancer Research Campaign.

### REFERENCES

Bird AP (1986) CpG-rich islands and the function of DNA methylation. *Nature* **321**: 209–213

Caldas C, Hahn SA, da Costa LT, Redston MS, Schutte M, Seymour AB, Weinstein CL, Hruban RH, Yeo CJ and Kern SE (1994) Frequent somatic mutations and homozygous deletions of the p16 (MTS1) gene in pancreatic adenocarcinoma [published erratum appears in *Nature Genet* 1994 Dec; **8(4)**: 410]. *Nature Genet* **8**: 27–32

Cleton-Jansen A-M, Collins N, Lakhani SR, Weissenbach J, Devilee P, Cornelisse CJ and Stratton MR (1995) Loss of heterozygosity in sporadic breast tumours at the BRCA2 locus on chromosome 13q12–q13. *Br J Cancer* **72**: 1241–1244

Collins N, McManus R, Wooster R, Mangion J, Seal S, Lakhani SR, Ormiston W, Daly PA, Ford D, Easton DF and Stratton MR (1995) Consistent loss of the wild type allele in breast cancers from a family linked to the BRCA2 gene on chromosome 13q12–13. *Oncogene* **10**: 1673–1675

Cornelis RS, Neuhausen SL, Johansson O, Arason A, Kelsell D, Ponder BA, Tonin P, Hamann U, Lindblom A, Lalle P, Longy M, Olah E, Scherneck S, Bignon YJ, Sobol H, Chang-Claude J, Larsson C, Spurr N, Borg A, Barkardottir RB, Narod S and Devilee P and the Breast Cancer Linkage Consortium (1995) High allele loss rates at 17q12–q21 in breast and ovarian tumors from BRCA1-linked families. The Breast Cancer Linkage Consortium. *Genes Chrom Cancer* **13**: 203–210

Cropp CS, Champeme MH, Lidereau R and Callahan R (1993) Identification of three regions on chromosome 17q in primary human breast carcinomas which are frequently deleted. *Cancer Res* **53**: 5617–5619

Devilee P, van den Broek M, Kuipers Dijkshoorn N, Kolluri R, Khan PM, Pearson PL and Cornelisse CJ (1989) At least four different chromosomal regions are involved in loss of heterozygosity in human breast carcinoma. *Genomics* **5**: 554–560

Foster KA, Harrington P, Kerr J, Russell P, DiCioccio RA, Scott IV, Jacobs I, Chenevix Trench G, Ponder BA and Gayther SA (1996) Somatic and germline mutations of the BRCA2 gene in sporadic ovarian cancer. *Cancer Res* **56**: 3622–3625

Futreal PA, Liu Q, Shattuck Eidens D, Cochran C, Harshman K, Tavtigian S, Bennett LM, Haugen Strano A, Swensen J, Miki Y, Eddington K, McClure M, Frye C, Weaver-Feldhaus J, Ding W, Gholami Z, Soderkvist P, Terry L, Jhanwar S, Berchuck A, Iglehart JD, Marks J, Ballinger D, Barrett JC, Skolnick M, Kamb A and Wiseman R (1994) BRCA1 mutations in primary breast and ovarian carcinomas. *Science* **266**: 120–122

Gessler M, Konig A, Arden K, Grundy P, Orkin S, Sallan S, Peters C, Ruyle S, Mandell J and Li F (1994) Infrequent mutation of the WT1 gene in 77 Wilms' tumors. *Hum Mutat* **3**: 212–222

Gonzalez Zulueta M, Bender CM, Yang AS, Nguyen T, Beart RW, Van Tornout JM and Jones PA (1995) Methylation of the 5′ CpG island of the p16/CDKN2 tumor suppressor gene in normal and transformed human tissues correlates with gene silencing. *Cancer Res* **55**: 4531–4535

Herman JG, Latif F, Weng Y, Lerman MI, Zbar B, Liu S, Samid D, Duan DS, Gnarra JR, Linehan WM and Baylin S (1994) Silencing of the VHL tumor-suppressor gene by DNA methylation in renal carcinoma. *Proc Natl Acad Sci USA* **91**: 9700–9704

Herman JG, Merlo A, Mao L, Lapidus RG, Issa JP, Davidson NE, Sidransky D and Baylin SB (1995) Inactivation of the CDKN2/p16/MTS1 gene is frequently associated with aberrant DNA methylation in all common human cancers. *Cancer Res* **55**: 4525–4530

Hosking L, Trowsdale J, Nicolai H, Solomon E, Foulkes W, Stamp G, Signer E and Jeffreys A (1995) A somatic BRCA1 mutation in an ovarian tumour (letter). *Nature Genet* **9**: 343–344

Kerangueven F, Allione F, Noguchi T, Adelaide J, Sobol H, Jacquemier J and Birnbaum D (1995) Patterns of loss of heterozygosity at loci from chromosome arm 13q suggests a possible involvement of BRCA2 in sporadic breast tumors. *Genes Chrom Cancer* **13**: 291–294

Lancaster JM, Wooster R, Mangion J, Phelan CM, Cochran C, Gumbs C, Seal S, Barfoot R, Collins N, Bignell G, Patel S, Hamoudi R, Larsson C, Wiseman RW, Berchuck A, Iglehart JD, Marks JR, Ashworth A, Stratton MR and Futreal PA (1996) BRCA2 mutations in primary breast and ovarian cancers. *Nature Genet* **13**: 238–240

Lekanne Deprez RH, Bianchi AB, Groen NA, Seizinger BR, Hagemeijer A, van Drunen E, Bootsma D, Koper JW, Avezaat CJ and Kley N (1994) Frequent NF2 gene transcript mutations in sporadic meningiomas and vestibular schwannomas. *Am J Hum Genet* **54**: 1022–1029

Li Y, Bollag G, Clark R, Stevens J, Conroy L, Fults D, Ward K, Friedman E, Samowitz W, Robertson M, Bradley P, McCormick F, White R and Cawthorn R (1992) Somatic mutations in the neurofibromatosis 1 gene in human tumors. *Cell* **69**: 275–281

Merajver SD, Pham TM, Caduff RF, Chen M, Poy EL, Cooney KA, Weber BL, Collins FS, Johnston C and Frank TS (1995) Somatic mutations in the BRCA1 gene in sporadic ovarian tumours. *Nature Genet* **9**: 439–443

Miki Y, Swensen J, Shattuck Eidens D, Futreal PA, Harshman K, Tavtigian S, Liu Q, Cochran C, Bennett LM, Ding W, Bell R, Rosenthal J, Hussey C, Tran T, McClure M, Frye C, Hattier T, Phelps R, Haugen-Strano A, Katcher H, Yakumo K, Gholami Z, Shaffer D, Stone S, Bayer S, Wray C, Bogden R, Dayanath P, Ward J, Tonin P, Narod S, Bristow P, Norris F, Helvering L, Morrison P, Rosteck P, Lai M, Barrett JC, Lewis C, Neuhausen S, Cannon-Albright L, Goldgar D, Wiseman R, Kamb A and Skolnick M (1994) A strong candidate for the breast and ovarian cancer susceptibility gene BRCA1. *Science* **266**: 66–71

Miki Y, Katagiri T, Kasumi F, Yoshimoto T and Nakamura Y (1996) Mutation analysis in the BRCA2 gene in primary breast cancers. *Nature Genet* **13**: 245–247

Nigro JM, Baker SJ, Preisinger AC, Jessup JM, Hostetter R, Cleary K, Bigner SH, Davidson N, Baylin S, Devilee P, Glover T, Collins FS, Weston A, Modali R, Harris C and Vogelstein B (1989) Mutations in the p53 gene occur in diverse human tumour types. *Nature* **342**: 705–708

Ohtani Fujita N, Fujita T, Aoike A, Osifchin NE, Robbins PD and Sakai T (1993) CpG methylation inactivates the promoter activity of the human retinoblastoma tumor-suppressor gene. *Oncogene* **8**: 1063–1067

Powell SM, Zilz N, Beazer Barclay Y, Bryan TM, Hamilton SR, Thibodeau SN, Vogelstein B and Kinzler KW (1992) APC mutations occur early during colorectal tumorigenesis. *Nature* **359**: 235–237

Shuin T, Kondo K, Torigoe S, Kishida T, Kubota Y, Hosaka M, Nagashima Y, Kitamura H, Latif F, Zbar B, Lerman M and Yao M (1994) Frequent somatic mutations and loss of heterozygosity of the von Hippel-Lindau tumor suppressor gene in primary human renal cell carcinomas. *Cancer Res* **54**: 2852–2855

Tang A, Varley JM, Chakraborty S, Murphree AL and Fung YK (1988) Structural rearrangement of the retinoblastoma gene in human breast carcinoma. *Science* **242**: 263–266

Takahashi H, Chiu HC, Bandera CA, Behbakht K, Liu PC, Couch FJ, Weber BL, LiVolsi VA, Furusato M, Rebane BA, Cardonick A, Benjamin I, Morgan MA, King SA, Mikuta JJ, Rubin SC and Boyd J (1996) Mutations of the BRCA2 gene in ovarian carcinomas. *Cancer Res* **56**: 2738–2741

Tavtigian SV, Simard J, Rommens J, Couch F, Shattuck Eidens D, Neuhausen S, Merajver S, Thorlacius S, Offit K, Stoppa Lyonnet D, Belanger C, Bell R, Berry S, Bogden R, Chen Q, Davis T, Dumont M, Frye C, Hattier T, Jammulapati S, Janecki T, Jiang P, Kehrer R, Leblanc JF, Mitchell J, McArthur-Morrison J, Nguyen K, Peng Y, Samson C, Schroeder M, Snyder S, Steele L, Stringfellow M, Stroup C, Swedlund B, Swensen J, Teng D, Thomas A, Tran T, Tranchant M, Weaver-Feldhaus J, Wong A, Shizuya H, Eyfjord J, Cannon-Albright L, Skolnick M, Weber B, Kamb A and Goldgar D (1996) The complete BRCA2 gene and mutations in chromosome 13q-linked kindreds (see comments). *Nature Genet* **12**: 333–337

Teng DH, Bogden R, Mitchell J, Baumgard M, Bell R, Berry S, Davis T, Ha PC, Kehrer R, Jammulapati S, Chen Q, Offit K, Skolnick MH, Tavtigian SV, Jhanwar S, Swedlund B, Wong AK and Kamb A (1996) Low incidence of BRCA2 mutations in breast carcinoma and other cancers. *Nature Genet* **13**: 241–244

Wooster R, Bignell G, Lancaster J, Swift S, Seal S, Mangion J, Collins N, Gregory S, Gumbs C, Micklem G, Barfoot R, Hamoudi R, Patel S, Rice C, Biggs P,

© Cancer Research Campaign 1997

Hashim Y, Smith A, Connor F, Arason A, Gudmundsson J, Ficence D, Kelsell D, Ford D, Tonin P, Bishop DT, Spurr N, Ponder BAJ, Eeles R, Peto J, Devilee P, Cornelisse CJ, Lynch H, Narod S, Lenoir GM, Egilsson V, Barkardottir RB, Easton DF, Bentley D, Futreal PA, Ashworth A and Stratton MR (1995) Identification of the breast cancer susceptibility gene BRCA2 (see comments). *Nature* **378**: 789–792

Yang Feng TL, Han H, Chen KC, Li SB, Claus EB, Carcangiu ML, Chambers SK, Chambers JT and Schwartz PE (1993) Allelic loss in ovarian cancer. *Int J Cancer* **54**: 546–551

Yoshiura K, Kanai Y, Ochiai A, Shimoyama Y, Sugimura T and Hirohashi S (1995) Silencing of the E-cadherin invasion-suppressor gene by CpG methylation in human carcinomas. *Proc Natl Acad Sci USA* **92**: 7416–7419

© Cancer Research Campaign 1997